# Is physical activity maintenance from adolescence to young adulthood associated with reduced CVD risk factors, improved mental health and satisfaction with life: the HUNT Study, Norway

**DOI:** 10.1186/1479-5868-9-144

**Published:** 2012-12-14

**Authors:** Vegar Rangul, Adrian Bauman, Turid Lingaas Holmen, Kristian Midthjell

**Affiliations:** 1 Faculty of Health Science, Nord-Trøndelag University College, Levanger, Norway; 2 HUNT Research Centre, Faculty of Medicine, Department of Public Health and General Practice, Norwegian University of Science and Technology, Levanger, Norway; 3 Prevention Research Collaboration, School of Public Health, University of Sydney, Sydney, Australia

## Abstract

**Background:**

Little is known about the effect maintaining physical activity throughout adolescence has on cardiovascular risk factors and health status in early adulthood. This ten-year prospective longitudinal study investigated whether differences in physical activity patterns from adolescence to young-adulthood showed different associations with subsequent cardio-metabolic risk factors and mental health in young-adulthood.

**Methods:**

Based on the second and third Norwegian Nord-Trøndelag Health Surveys (HUNT2 and 3), we included 1869 individuals (838 males) participating in Young-HUNT (1995–97), aged 13–19 years and followed-up at HUNT3 (2006–08), aged 23–31. Self-reported physical activity (PA), mental health and perceived health were recorded, along with measurements of body mass index (BMI), waist circumference (WC), total cholesterol (TC), HDL cholesterol, glucose, triglycerides, resting heart rate (HR) and blood pressure. We used separate linear regressions models to investigate associations between physical activity and each CVD risk factor, and logistic regression analysis to examine PA patterns and subsequent mental health. Physically active maintainers were compared to inactive maintainers. Adopters (inactive as adolescents and physically active as young adults) were compared to inactive maintainers and to those who discontinued activity (relapsers).

**Results:**

Active maintainers had significantly lower HR, compared to all other PA patterns. Active maintaining men had significantly lower WC than relapsers and inactive maintainers. When adjusted for age and gender, WC, BMI, HR, diastolic blood pressure and HDL-C showed significant differences comparing active maintaining to other PA patterns. Comparing inactive maintainers against adopters, only HR was significantly lower. Male adopters did not differ significantly in CVD risk compared to inactive maintainers and relapsers. Among females adopting was associated with lower HR and TC compared to inactive maintainers. Active maintainers showed better mental health than inactive maintainers. Active maintaining males had an increased likelihood of good mental health compared to adopters. Active maintaining females reported greater satisfaction with life compared to adopters.

**Conclusions:**

Those who maintained their physical activity from adolescence to young adulthood demonstrated a significantly lower CVD risk and better mental health, compared to inactive maintainers. Compared to inactivity maintainers and relapsers, adopting physical activity was not significantly associated with lowered CVD risk. Adopting physical activity between adolescence and young adulthood may not necessarily protect against mental distress.

## Background

It is well documented that physical activity is associated with numerous health benefits, both in youth and adulthood. Several observational studies suggest that being physically active is associated with lowered risk for cardiovascular disease (CVD), certain cancers and improved mental health and quality of life [1]. There is also evidence for a dose–response relationship between physical activity and health outcomes [2]. The literature shows a consistent, but moderate association between physical activity in adolescence and adulthood [3]. This modest association is partly due to the complex nature of physical activity behaviour, and also due to a paucity of population tracking studies.

Physical activity is important in cardiovascular disease prevention, mediated through a direct disease protective effect, and through its association with favourable cardiovascular risk factor profiles [4,5]. Cardiovascular disease (CVD) is the leading cause for morbidity and mortality worldwide [6], and risk factors for CVD include obesity, smoking, low levels of high-density lipoprotein (HDL-C), high level of total cholesterol (TC), triglycerides and high blood pressure (BP) [7,8]. In addition, physical activity has a role in psychosocial health and in promoting good mental health [9], although much of this prospective epidemiological evidence was established in adult populations [10].

The present study focuses on adolescence, as an important period for healthy lifestyle habit development, and for maintaining low risk cardiovascular profiles and improving mental health [11-13]. Physical activity habits may decline in adolescence and in the transition to adulthood, and the health implications of this are important for chronic disease prevention efforts [14,15].

To date, few studies have examined the relationship between physical activity in adolescence and subsequent CVD risk and mental health in adulthood. Previous studies examining physical activity and physical fitness, found that physical fitness, but not physical activity was associated with lowered CVD risk, but often used small non-representative samples [7,16,17]. Sassen at al. reported that physical fitness and to a smaller degree physical activity were inversely associated with CVD risk (clustering) [18]. The health benefits of physical activity, not just fitness, on adolescent and young adult health risk factors require further investigation [19].

For mental health, most studies report relationships between physical activity and mental health using short follow-up periods. Little is known about the long-term effects of adolescent physical activity on adult mental health. Physical activity reported at least of moderate intensity seems to be associated with better mental health [20].

To our knowledge, no previous longitudinal population based studies have studied the physical activity pattern from adolescence to young adulthood as predictor of cardiovascular disease risk factors and poor mental health in young adulthood. This information is important from a preventive health perspective.

The purpose of this ten-year prospective longitudinal study analysis was to investigate:

- Are those who maintain physically active behaviour (active maintainers) different in terms of (i) cardio-metabolic risk factors, and (ii) mental health outcomes compared to those that maintained an inactive behaviour (inactive maintainers)?

- Are active maintainers (AM) different to all other groups: those who maintained an inactive behaviour (IM), those who adopted more physical activity behaviour (adopters), or those who relapsed to a low active state (relapsers)?

- Specifically; is adopting physical activity health-enhancing, by comparing cardio-metabolic risk and mental health between adopters and active maintainers; and between adopters and less active patterns (relapsers and inactive maintainers)?

## Material and methods

### Study population

In 1995–97 all inhabitants (approximately *n* = 127 000) aged 13 years and older were invited to participate in the second population survey in the county of Nord-Trøndelag in Norway, the Nord-Trøndelag Health Study (HUNT), http://www.ntnu.no/hunt/english.

A total of 8983 adolescents (90% response rate) participated in the youth component (13–19 years old) of the study (Young-HUNT1). These participants were also invited 10 years later to participate in the adult part of the HUNT3 survey which was carried out in 2006–08. We followed a sub-sample of 2172 who had participated in both surveys. We excluded 303 persons from the analysis because they had insufficient blood for analysis. In the present analysis, the study cohort sample comprised 1869 individuals (n = 838 male) who participated in both Young-HUNT1, aged 13–19 years old (baseline, T1) and HUNT3, aged 23–31 (follow-up, T2).

The population is stable, and the sex and age distribution fairly similar to that of Norway as a whole, except slightly lower average income and education [21].

### Measures

In Young-HUNT1 participants completed a self-administrated questionnaire during one school session and participated in a clinical examination that included measurement of height and weight at school. In HUNT3, they completed the questionnaires at home and had a clinical examination and collection of blood samples at screening stations. A detailed description of the HUNT Study is reported elsewhere [21,22].

#### Physical activity

Physical activity in adolescence was assessed by questions used in the World Health Organization Health Behaviour in School-Aged Children (WHO HBSC) Surveys [5], and in adulthood by leisure time PA questionnaire. The questions record the responder’s PA in sports or exercise, asking the number of days a week they were physically active at a moderate to vigorous intensity (MVPA) in their leisure time. The question was: “Outside school hours: How many days a week do you play sports or exercise so that you get out of breath or sweat?”. The response alternatives were: “every day”, “4-6 days a week”, “2-3 days a week”, “one day a week”, “not every week, but at least once every 14th day”, “not every 14th day, but at least once a month”, “less than once a month” and “never” [23]. The adults question asks “How often do you exercise?”. The response alternatives were: “never”, “less than once a week”, “once a week”, “2–3 times a week” and “nearly every day”. Both questionnaires were dichotomised into; “inactive” if response was <2-3 days/week (adolescence) or <2-3 times a week (adulthood) and “active” if response was ≥2-3 days/week or ≥2-3 times a week. Both questionnaires have been validated against physical fitness (VO_2peak_) and PA level (by ActiReg®), among adolescents and men between 20–39 years old in Nord-Trøndelag [24-26].

Based on these physical activity categories, we constructed a measure of the pattern of physical activity from adolescence to young adulthood. Those who were active at both time points, were “active maintainers” (AMs), those who had been active and became inactive were described as “relapsers”. Those who moved from being inactive to active, were classified as “adopters”, and those who were inactive during adolescence and still inactive at young adulthood were “inactive maintainers” (IMs) [13].

#### Metabolic measures (CVD risk factors)

A non-fasting blood sample was drawn from all participants at follow-up (HUNT3). Serum samples were analysed for total cholesterol (TC), high-density lipoprotein cholesterol (HDL-C), glucose and triglycerides. The TC was analysed by enzymatic cholesterol esterase methodology, applying Reagent kit 7D62-20 cholesterol. HDL-C was analysed by accelerator selective detergent methodology, applying Reagent kit 3 K33-20 Ultra HDL. Non-fasting glucose was analysed by Hexokinase/G-G-PDH methodology, applying Reagent kit 3 L82-20 Glucose. Triglycerides was analysed by Glycerol Phosphate Oxidase methodology, Reagent kit 7D74 Triglyceride. All reagent kits were from Abbott (Clinical Chemistry, USA) and the samples were measured in mmol/L.

#### Waist circumference and body mass index

Both in Young-HUNT1 and HUNT3, trained nurses measured the participants’ height and weight following the same standardised procedures, participants wearing light clothes and without shoes. Body mass index (BMI) was calculated as weight divided by height squared (kg/m^2^). Waist circumference was measured to the nearest 1.0 cm using a non-elastic measuring tape, assessed after maximal expiration, and measured at the umbilicus or midway between the subcostal margin and the iliac crest if the latter was largest.

#### Blood pressure and heart rate

In HUNT3, trained nurses measured blood pressure (BP) in seated participants, with a Dinamap 845XT (Criticon, Florida, USA) based on oscillometry. Blood pressure was measured automatically three times at one minute intervals. The arithmetic mean of the second and third systolic and diastolic blood pressure readings were used in this study. The resting heart rate (HR) was measured by the Dinamap, and expressed as beats/min.

#### Mental health and perceived health status/satisfaction with life

Mental health was measured with the Cohort of Norway Mental Health Index (CONOR-MHI). The CONOR-MHI includes seven questions asking about psychosocial distress, and is modified from the General Health Questionnaire [27] and the Hopkins Symptom Checklist [28]. The CONOR-MHI has been shown to be a valid measure of mental health status encompassing both anxiety and depression [29]. The 7 questions in the CONOR-MHI were: “Have you, in the last 2 weeks, felt; 1) nervous and restless; 2) troubled by anxiety; 3) confident and calm; 4) irritable; 5) happy and optimistic; 6) down/depressed; or 7) lonely?” Each item has four answer categories, ranging from “no”, “a little, “moderately” and “very much”, given the values 1–4. Data on items 3 and 5 were reversed in the analysis. The index is based on means of the questions and calculated by dividing the total score on seven (number of items) and 4 (4-point scale). The CONOR-MHI results were analyzed as both continuous and categorical variables. As a categorical score, we divided the summary score distribution into tertiles (scores of 1.00–1.79, 1.80–2.29 and 2.30–6.00 respectively). Five of the 7 questions (item 1, 2, 4, 6 and 7) were also analyzed separately in logistic regression models. The answers were recorded into two categories, and the outcomes were “no” and “a little, moderately and very much”.

Current subjective health was self-rated as “poor”, not so good”, “good” or “very good”. In logistic regression analysis this variable was recorded into two categories, combining “poor and not so good health” and “good and very good health”. The participants reported their ‘satisfaction with life’, measured by a question asking: “Thinking about your life at the moment, would you say that you by and large are satisfied with life, or are you mostly dissatisfied?”. Responses were recorded into two categories, 1 “dissatisfied”, including “a bit of both”, “somewhat dissatisfied”, “dissatisfied” and “very dissatisfied” and 2 “satisfied” including “satisfied” and “very satisfied”.

### Ethics

Participants and the parents or legal guardians of adolescents under the age of 16 years, signed a written consent to take part in the study. The study was approved by the Regional Committee for Medical Research Ethics and the Norwegian Data Inspectorate Board.

### Statistical analysis

The participants’ characteristics were calculated as means (±standard deviation) and percentages. We applied ANOVA with Scheffé’s method for post-hoc contrasts to test the differences between means. We present p-values (significance level p < 0.05) and F-statistics from these analyses. This is a flexible and conservative post hoc procedure, and is a preferable method for comparisons that involve contrasts of more than two means at a time. We used separate linear regression models to investigate associations between physical activity and each of the different CVD risk factors. First, physically active maintainers (AMs) were compared to inactive maintainers (IMs), unadjusted and adjusted for age and gender, to examine the linear relationships between CVD risk factors and physical activity maintenance. Second, we grouped relapsers, adopters and IMs by comparing them against AMs to investigate the relationship with CVD risk factors. In addition, we also separately examined the linear relationships by comparing adopters against AMs and adopters against the common group of IMs and relapsers. We also combined IMs and relapsers, and compared them with AMs and investigated the associations with CVD risks factors and mental health (not shown in tables). On the basis of these analyses, we stratified analyses by gender and did the same analyses stratified by gender.

In our linear regression models, we compared those who became physically active (adopters) against those who were physically inactive (inactive maintainers), to test the hypothesis that increasing physical activity is associated with health benefits compared to those remaining inactive. We also combined IMs and relapsers and compared them with adopters to further investigate these associations.

To examine the relationship between physical activity patterns and mental health and satisfaction with life we used multiple binary logistic regression analyses, in separate models. The analysis compared physical activity patterns from adolescence to young adulthood (inactive maintainers (IMs) vs. active maintainers (AMs) and IM vs. adopters) and outcome (perceived health, satisfaction with health and mental health status) at follow-up. We also combined IMs and relapsers and compared them with AMs (not shown in tables). Gender specific analyses were performed, and all analyses were age adjusted. All statistical analyses were performed with IBM SPSS (SPSS Inc., Chicago IL, USA) version 19.1.

## Results

A total of 1869 participated (males 42.8%), with a mean age of 16 years at baseline (adolescence) and 27 years at follow-up (adulthood). Participants’ characteristics are listed in Table 1, stratified by gender. Four patterns of physical activity from adolescence to young adulthood emerged: active maintainers (AMs)(42.3%, males 38.1%, females 45.4%), reporting high PA both in adolescence and early adulthood; relapsers (25.4%, males 31.4%, females 21.3%), showing high PA in adolescence, but low PA in adulthood; adopters (14.2%, males 11.4%, females 16.3%), changing from low PA in adulthood to high PA as young adults; and inactive maintainers (IMs)(18%, males 17%, females 19.1%), classified with low PA at both time points.

**Table 1:** Participants characteristics at baseline and follow up (N = 1869)

**Variables**	**Males (*n*** **= 799)**							**Females (*n*** **= 1070)**						
	**AM*n*** **= 304 (38.1%)**	**Relapsers*n*** **= 251 (31.4%)**	**Adopter*n*** **= 91 (11.4%)**	**IM*n*** **= 153 (19.1%)**				**AM*n*** **= 486 (45.4%)**	**Relapsers*n*** **= 228 (21.3%)**	**Adopter*n*** **= 174 (16.3%)**	**IM*n*** **= 182 (17.0%)**			
	**Mean (SD)**	**Mean (SD)**	**Mean (SD)**	**Mean (SD)**	***F***	***p***	**Scheffe Post hoc test**	**Mean (SD)**	**Mean (SD)**	**Mean (SD)**	**Mean (SD)**	***F***	***p***	**Scheffe Post hoc test**
**Baseline measures**														
Age	15.8 (1.84)	15.8 (1.77)	16.1 (1.81)	16.3 (1.79)	3.17	.024		15.8 (1.83)	15.8 (1.75)	16.1 (1.85)	16.5 (1.83)	6.02	.000	IM > rel., AM
BMI	20.8 (2.96)	20.9 (3.29)	21.7 (3.41)	21.4 (3.47)	2.47	.061		21.4 (3.20)	21.3 (2.94)	21.6 (3.20)	21.5 (3.39)	.20	.896	
**Follow-up measures**														
Age	27.0 (1.92)	27.0 (1.88)	27.5 (1.94)	27.6 (1.96)	4.29	.005	IM > rel., AM	27.0 (1.94)	27.0 (1.79)	27.3 (1.99)	27.7 (1.90)	5.59	.001	IM > rel., AM
Sitting time (hour/day)	6.0 (2.92)	6.1 (3.13)	6.5 (3.16)	5.9 (3.07)	.77			.509	5.7 (2.45)	5.1 (2.65)	5.7 (2.71)	5.8 (2.81)	2.50	.059	
Waist circumference (cm)	89.7 (9.70)	92.7 (12.28)	91.8 (11.21)	93.4 (12.38)	4.84	.002	AM < rel., IM	84.8 (12.90)	85.9 (12.97)	87.0 (13.42)	87.3 (13.20)	2.03	.108		
BMI	25.8 (3.70)	26.4 (4.90)	26.5 (4.02)	26.4 (4.49)	1.49			.217	25.0 (4.72)	25.3 (4.74)	25.8 (5.07)	25.6 (4.91)	1.18	.316	
Resting heart rate	66.1 (10.74)	71.0 (11.47)	72.3 (12.67)	71.6 (10.43)	14.32	.000	AM < IM, rel., adopt.	70.2 (10.19)	74.8 (11.08)	73.2 (9.97)	76.7 (10.43)	21.10	.000	AM < IM, rel., adopt.	
Diastolic blood pressure (mmHg)	67.1 (8.74)	68.4 (8.64)	68.6 (8.51)	67.5 (9.54)	1.17	.320		64.1 (7.92)	63.9 (7.90)	66.0 (7.68)	66.1 (7.75)	4.53	.004	AM < IM	
Systolic blood pressure (mmHg)	127.0 (10.00)	127.6 (11.44)	128.0 (11.93)	125.6 (11.09)	1.13	.336		114.6 (10.52)	114.7 (9.03)	116.1 (10.14)	115.9 (10.92)	1.34	.259		
HDL-cholesterol (mmol/l)	1.20 (.25)	1.13 (.26)	1.13 (.25)	1.14 (.24)	5.05	.002	AM > rel.	1.45 (.32)	1.43 (.35)	1.42 (.33)	1.38 (.30)	1.62	.184		
Cholesterol (total) (mmol/l)	4.66 (.92)	4.87 (1.00)	4.68 (1.03)	4.90 (1.02)	3.18	.023		4.70 (.88)	4.90 (1.19)	5.01 (1.05)	4.74 (.96)	4.96	.002	AM < adopt.	
Glucose (mmol/l)	5.02 (.91)	5.17 (1.13)	5.37 (3.03)	5.07 (.88)	1.64	.179		4.99 (1.41)	4.83 (.73)	4.80 (.59)	4.86 (.65)	1.91	.126		
Triglycerides	1.55 (1.01)	1.81 (1.19)	1.73 (1.05)	1.70 (1.11)	2.06	.104		1.13 (.72)	1.23 (.66)	1.24 (.77)	1.21 (.58)	1.37	.250		

*IM* = inactive maintainers, *AM* = active maintainers, *rel* = relapsers, *adopt* = adopters.

Differences by gender were significant for AMs and relapsers.

The mean age was significantly higher, for both genders, among inactive maintainers (IMs) compared to relapsers and active maintainers at follow-up (Table 1). The same pattern was seen at baseline in women.

Mean resting heart rate was significantly lower in the AM group, compared to the three other PA patterns, for both genders. Among men, the AM had significantly lower waist circumference than relapsers and IM, and the AM group had significantly higher HDL-C compared to relapsers. Among females, the AMs had significantly lower total cholesterol (TC) than adopters.

Linear regression analysis in Additional file 1 shows the relationship between the patterns of PA and CVD risk factors. Unadjusted, all variables except systolic blood pressure and glucose were significantly better in the AMs than the IMs group (Additional file 1). When adjusted for age and gender, the AM showed significant, slightly smaller waist circumference (WC), lower resting HR and higher HDL-C compared to the IMs (Additional file 1). In the additional analysis, comparing the remaining groups (combined IM + relapsers + adopters) against the AM, we found additional significant differences for TC, triglyceride levels and BMI (right hand column, Additional file 1)

Additional file 2 presents data on those who adopted physical activity, compared to active maintainers (AMs) in the left hand column, and against all hypothetically ‘less active categories’, relapsers and inactive maintainers (IMs), shown in the right hand columns. Compared to adopters, the adjusted analyses showed that AMs had a significantly lower waist circumference, heart rate, diastolic BP and total cholesterol. However, compared to the IM + relapsers, the adopters showed no significant differences on any of the cardiovascular risk parameters.

Table 2 shows the gender specific associations between patterns of physical activity and cardiovascular risk factors. For males, AMs had more favourable risk factor profiles for WC, HR and HDL-C, compared to inactive maintainers. Among females, only HR was significantly lower in this comparison. Comparing adopters against AMs, for males, the AM had a lower HR, and higher HDL-C, and for women the AM had a lower HR and diastolic BP. Examining the AM against all others (Table 3), males showed significantly favourable BMI, WC, HR and HDL-C, and females showed favourable WC, HR and diastolic BP. Adopters did not show a favourable profile compared to inactive maintainers and relapsers for males, and only HR was lower among adopting females (right hand columns, Table 3).

**Table 2:** CVD risk in inactive maintainers and adopters compared to active maintainers in young adulthood

**Variables**	IMs against AMs^*^			IMs against AMs^*^			**Adopters against AMs**^**#**^			**Adopters against AMs**^**#**^		
	**Males**			**Females**			**Males**			**Females**		
	**B**	***P***	**95% CI**	**B**	***P***	**95% CI**	**B**	***P***	**95% CI**	**B**	***P***	**95% CI**
BMI	-.58	.146	−1.37, 0.20	-.34	.418	−1.16, 0.48	-.69	.129	−1.59, 0.20	-.66	.123	−1.49, 0.18
Waist circumference (cm)	−3.54	.001	−5.64, -1.43	−1.89	.103	−4.17, 0.38	−1.87	.124	−4.25, 0.51	−1.90	.102	−4.19, 0.39
Resting heart rate (HR)	−5.66	.000	−7.75, -3.56	−6.25	.000	−8.02, -4.48	−6.21	.000	−8.87, -3.54	−2.82	.002	−4.57, -1.07
Diastolic blood pressure (mmHg)	.03	.971	−1.85, 1.92	−1.77	.016	−3.20, -0.33	−1.17	.285	−3.33, 0.98	−1.79	.017	−3.25, -0.33
Systolic blood pressure (mmHg)	1.65	.144	−0.57, 3.86	−1.22	.218	−3.17, 0.72	-.80	.551	−3.43, 1.84	−1.51	.127	−3.46, 0.43
HDL-cholesterol (mmol/l)	.06	.018	0.01, 0.11	.05	.069	−0.00, 0.11	.07	.020	0.01, 0.13	.02	.423	−0.03, 0.08
Cholesterol (total) (mmol/l)	-.19	.046	−0.38, -0.00	-.03	.706	−0.19, 0.13	.01	.967	−0.22, 0.23	-.30	.000	−0.46, -0.14
Glucose (mmol/l)	-.03	.751	−0.21, 0.15	.16	.156	−0.06, 0.38	-.33	.108	−0.72, 0.07	.21	.070	−0.02, 0.43
Triglycerides	-.12	.308	−0.36, 0.11	-.09	.224	−0.23, 0.05	-.17	.256	−0.45, 0.12	-.11	.161	−0.27, 0.04

Adjusted for age.

^*^ Inactive maintainers (IMs) vs active maintainers (AMs).

^#^ adopters vs active maintainers (AM).

**Table 3:** Different physical activity patterns in relation to CVD risk in Young adulthood

**Variables**	IMs + relap + adopt against AMs^*^			IMs + relap + adopt against AMs^*^			**Adopters against IMs + relap**^**#**^			**Adopters against IMs + relap**^**#**^		
	**Males**			**Females**			**Males**			**Females**		
	**B**	***P***	**95% CI**	**B**	***P***	**95% CI**	**B**	**P**	**95% CI**	**B**	**P**	**95% CI**
BMI	-.64	.042	−1.26, 0.02	-.45	.130	−1.04, 0.13	-.11	.838	−1.17, 0.95	-.33	.455	−1.21, 0.54
Waist circumference (cm)	−2.95	.000	−4.56, -1.33	−1.63	.047	−3.24, -0.02	1.22	.389	−1.56, 4.00	-.51	.675	−2.91, 1.89
Resting heart rate (HR)	−5.31	.000	−6.91, -3.71	−4.66	.000	−5.93, -3.39	−1.03	.441	−3.64, 1.59	2.44	.011	0.55, -4.32
Diastolic blood pressure (mmHg)	-.89	.188	−2.21, 0.44	−1.05	.045	−2.07, 0.03	-.32	.768	−2.46, 1.81	−1.06	.162	−2.55, 0.43
Systolic blood pressure (mmHg)	.08	.929	−1.59, 1.75	-.91	.180	−2.24, 0.42	−1.09	.443	−3.89, 1.70	-.87	.368	−2.77, 1.03
HDL-cholesterol (mmol/l)	.07	.000	0.04, 0.11	.03	.123	−0.01, 0.07	.01	.874	−0.05, 0.06	-.01	.757	−0.07, 0.05
Cholesterol (total) (mmol/l)	-.17	.021	−0.31, -0.03	-.18	.004	−0.30, -0.06	.21	.083	−0.03, 0.44	-.17	.087	−0.37, 0.03
Glucose (mmol/l)	-.15	.152	−0.35, 0.05	.17	.013	0.04, 0.30	-.23	.230	−0.60, 0.15	.05	.429	−0.07, 0.17
Triglycerides	-.21	.025	−0.39, -0.03	-.11	.038	−0.20, 0.01	.05	.774	−0.26, 0.36	-.01	.850	−0.16, 0.13

Adjusted for age.

^*^Active maintainers (AMs) vs inactive maintainers (IMs) + relapsers + adopters.

^#^ Adopters vs inactive maintainers (IMs) + relapsers.

Mental health associations are shown in Table 4. The AMs reported better self-rated health status and lower mental health CONOR scores than IMs. In addition, female AMs showed a twofold likelihood of being satisfied with life, and a reduced risk of feeling nervous or troubled by anxiety compared to IM. Compared to adopters (Table 4, right columns), male AMs showed an increased likelihood of good health status, satisfaction with life, and a lower likelihood of reporting high CONOR scores or depression. For females, AM only showed greater life satisfaction, compared to adopters.

**Table 4:** Satisfaction with life and mental health in inactive maintainers and adopters against active maintainers

**Variables**	**IMs against AMs**						**Adopters against AMs**					
	**Males**			**Females**			**Males**			**Females**		
	**OR**	**95% CI**	***P***	**OR**	**95% CI**	***P***	**OR**	**95% CI**	***P***	**OR**	**95% CI**	***P***
Perceived health												
Not so good	1.0			1.0			1.0			1.0		
Good/very good	2.87	1.32, 6.23	.008	2.59	1.51, 4.46	.001	3.60	1.54, 8.41	.003	1.59	.86, 2.91	.138
Satisfaction with life												
Dissatisfied	1.0			1.0			1.0			1.0		
Satisfied	1.81	.98, 3.38	.060	1.96	1.22, 3.14	.005	2.15	1.08, 4.29	.029	1.88	1.17, 3.01	.009
Mental health status (CONOR-MHI)*												
First tertile (1.00-1.79)	1.0			1.0			1.0			1.0		
Second tertile (1.80-2.29)	.44	.27, .73	.001	.47	.30, .75	.001	1.06	.59, 1 .88	.857	.98	.65, 1.48	.919
Third tertile (2.30-6.00)	.26	.15, .48	.000	.42	.26, .70	.001	.39	.21, .74	.004	1.01	.63, 1.62	.960
Feel Nervous/restless												
No	1.0			1.0			1.0			1.0		
Little, good amount, very much	.25	.52, 1.19	.252	.62	.44, .88	.007	.63	.39, 1.03	.066	.95	.66, 1.36	.775
Felt troubled by anxiety												
No	1.0			1.0			1.0			1.0		
Little, good amount, very much	.59	.33, 1.06	.079	.47	.29, .75	.002	.64	.32, 1.30	.218	.52	.49, 1.44	.519
Felt down/depressed												
No	1.0			1.0			1.0			1.0		
Little, good amount, very much	.73	.47, 1.14	.162	.70	.49, 1.00	.054	.53	.32, .88	.014	1.23	.84, 1.81	.282

Adjusted for age.

*CONOR-MHI (Cohort of Norway Mental Index) score in tertiles.

Table 5 shows the comparisons between AMs and all less active patterns, and showed mental health advantage for AMs, especially for males. These were less strong among females, but AMs showed reduced anxiety compared to other patterns. There was very little difference between adopters and the less active patterns (right hand columns), except for the middle CONOR category, for males.

**Table 5:** The likelihood of physical activity patterns associated with satisfaction with life and mental health

**Variables**	**IMs + relapsers + adopters against AMs**						**Adopters against IMs + relapsers**					
	**Males**			**Females**			**Males**			**Females**		
	**OR**	**95% CI**	***P***	**OR**	**95% CI**	***P***	**OR**	**95% CI**	***P***	**OR**	**95% CI**	***P***
Perceived health												
Not so good	1.0			1.0			1.0			1.0		
Good	3.07	1.62, 5.84	.001	2.04	1.32, 3.16	.001	1.27	.64, 2.52	.494	.75	.42, 1.31	.308
Satisfaction with life												
Dissatisfied	1.0			1.0			1.0			1.0		
Satisfied	1.77	1.10, 2.86	.019	1.75	1.23, 2.50	.002	1.29	.69, 2.42	.420	1.14	.72, 1.81	.578
Mental health status (CONOR-MHI)*												
First tertile (1.00-1.79)	1.0			1.0			1.0			1.0		
Second tertile (1.80-2.29)	.55	.39, .76	.000	.78	.58, 1.04	.088	2.23	1.26, 3.94	.006	1.39	.91, 2.13	.132
Third tertile (2.30-6.00)	.38	.25, .58	.000	.72	.52, 1.00	.050	1.04	.57, 1.89	.895	1.62	1.00, 2.65	.052
Feel Nervous/restless												
No	1.0			1.0			1.0			1.0		
Little, good amount, very much	.76	.56, 1.03	.074	.80	.63, 1.03	.087	.81	.51, 1.29	.376	1.24	.86, 1.79	.253
Felt troubled by anxiety												
No	1.0			1.0			1.0			1.0		
Little, good amount, very much	.79	.50, 1.26	.322	.63	.44, .92	.016	.80	.41, 1.56	.515	1.44	.85, 2.44	.180
Felt down/depressed												
No	1.0			1.0			1.0			1.0		
Little, good amount, very much	.70	.50, .97	.034	.78	.75, 1.25	.784	.67	.42, 1.10	.112	1.40	.95, 2.06	.091

Adjusted for age.

*CONOR-MHI (Cohort of Norway Mental Index) score in tertiles.

In linear regression models using the CONOR score as continuous, we found similar associations where the AM had a significant lower CONOR-MHI score than the IMs, in both genders (males β = −0.26; 95%CI:-0.39- 0.13; *p < .000,* females β = −0.25; 95%CI:-0.37- -0.12; *p* < .000).

We also conducted two additional analyses. First, to test the PA change with a higher cut off for physical activity, and the second we analysed when change of PA among adopters and relapses occurred.

We re-analysed the data with a higher cut off for physical activity; “≥4-6 days/week or nearly every day” instead of “≥2-3 days/week or ≥2-3 times a week”. This did not change our findings. Measuring physical activity four years after baseline, thus being able to see when the change of PA behaviour occurred both for adopters and relapsers, revealed that nearly the same number of people changed their PA early and late in this ten-year period.

The difference was less than 6%, while among adopters 44% became active between years five and ten, and 51% of relapsers became inactive between years five and ten.

## Discussion

This population based longitudinal study explored differences in physical activity patterns between adolescence and young adulthood were associated with cardiovascular disease (CVD) risk factors and mental health in young adulthood. To our knowledge no other study of this size has previously examined tracking of PA associated with CVD risk factors and mental health.

To characterize participants, we compared four physical activity patterns; active maintainers (AMs), adopters, relapsers and inactive maintainers (IMs), observed over a ten-year period. In adolescence (baseline) and in young adulthood (follow-up) there were no significant differences (mean) among these physical activity groups and overweight/obesity measured as BMI. This is in accordance with studies on the relationship between physical activity and obesity measured as BMI, who reported that the relationship may be more strongly related to cardiorespiratory fitness or screen activities (watching television, computer use) than physical activity [30,31]. But the obesity-related indicator, waist circumference (WC) was lower among AM males than relapsers and IMs. This is supported by Lakerveld et al., who found that abdominal obesity was associated with reduced physical activity over a period of five years [32]. Analyses stratified by gender showed that the association between PA and WC was only significant in males. An inverse relationship between physical activity and overweight/obesity is well known [33,34], although few studies have examined males and females separately.

We found no association between PA behaviour and WC, comparing adopters and IMs + relapsers, even in an unadjusted analysis. Overweight/obesity in adulthood has been associated with decreased probability of being physically active [35]. The lack of association between the adopters and WC in our study, contrasts with the assumption that improvement in physical activity during this time period reduces subsequent obesity and CVD risk [36].

Physical inactivity is a well-known CVD risk factor in adolescence [37], and patterns of physical activity maintenance may be protective in longitudinal studies. This was confirmed when we compared the two extreme groups (IMs compared to AMs); AMs had a much better CVD risk profile in adulthood than the IMs, both in the unadjusted and adjusted analyses, and supporting other studies reporting that physical activity and physical fitness may protect against CVD risk [17]. We found a significant gender difference in the relationship between PA and CVD risk factors. Resting heart rate was lower for AMs in both sexes, but AM males had a considerably smaller waist circumference (WC), lower TC (triglyceride concentration) and higher HDL cholesterol (HDL-C) than the IMs. The gender difference could be anticipated for TC, where previous population studies have found a higher TC in men compared women in the first fifty years [38]. There is also some evidence that a high level of TC is a significant independent risk factor for CVD for both genders [38]. HDL-C is an independent predictor of CVD, both in males and females, but females may have a lower HDL-C than males [39]. Our results show an opposite gender difference, female IMs did not have a higher risk for low HDL-C than AMs. However, it is still important to focus on TC and HDL-C level, in both sexes among those who are not physically active.

The PA behaviour and CVD risk may be confounded by obesity, which we know is associated with higher triglyceride levels and cholesterol. But our descriptive data (Table 1) indicated no significant differences in BMI between our four physical activity patterns. This lack of difference in BMI between the different PA patterns is interesting, especially when the patterns did show differences for CVD risks. Additional analyses examined how obesity affected the associations, by adjusting for obesity measured as BMI, both at baseline and follow-up (data not shown). These analyses did not attenuate the results presented in the tables, indicating that physical activity patterns and subsequent CVD risk are likely to be independent of obesity.

Several cross-sectional studies have shown a positive effect by physical activity on mental health in general, and in particularly on self-perception and self-esteem [40]. Physical activity has also been recommended as a tool in therapy for depression and anxiety [41], but information on how different physical activity behaviours from adolescence to young adulthood affects mental health and satisfaction with life in adulthood is sparse. Our longitudinal data indicate that the AMs had better life satisfactions and mental health status than inactive maintainers. In addition, there were gender differences, with female AMs having a lower likelihood of feeling nervous/restless and being troubled by anxiety, compared to inactive maintainers. We did not see this in males, but the odds ratios indicate the same trend also for males. This is in accordance with previous studies, where physically active adults’ reports fewer symptoms of anxiety than physically inactive persons [42]. Some longitudinal studies have also found negative associations between sedentary behaviour and mental health, while sedentary behaviour as TV viewing was associated with increased odds of mental distress [43].

Physical activity is important for maintaining good health, and physically inactive people have a higher incidence of cardiovascular disease [36]. In addition to its preventive effect, physical activity is also recommended in treatment of several chronic diseases [3]. It could therefore be expected that those who increased their physical activity from adolescence to adulthood (adopters) might have a lowered CVD risk and better mental health than those who stayed inactive (IMs) or relapsed to lower physical activity. Our data indicated, however, that the adopters had no metabolic or mental health advantages compared to IMs and relapsers. This is rather surprising, because we would expect that increased physical activity would have a positive effect on risk factors. One explanation could be that their PA increase was minor and occurred late in the measured period. According to the literature [44], we expected that the change of PA would take place in adolescence (years zero to four in the ten-year period) but our data on adopters and relapsers did not support this. In additional analyses we also compared these two groups (adopters and relapsers) according to when they changed the PA in this period. They did not differ in CVD risk factors. The higher cut point for physical activity did not change our findings.

The unexpected findings on adopters compared to inactive maintainers and relapsers (Table 4–5), became more apparent when we compared adopters with AMs. The active maintainers had a better CVD risk profile in adulthood than the adopters, allowing us to combine the adopters, IMs and relapsers in one group. AMs differed from the other groups concerning association with CVD risk factors. We also revealed the same tendency on the likelihood of physical activity patterns associated with mental health and satisfaction with life. A physiologically plausible explanation could be that adopters altered their physical activity pattern late in the period, and we therefore cannot distinguish them from IMs. Our additional analysis shows, however that it mainly changed early in the adolescence in our 10-year follow-up. The adopter group was also smaller than the other groups, which could also be an explanation for the unexpected absence of positive outcome of adopting PA. But, in the descriptive data the adopter group is quite similar to the IMs and relapsers (Table 1).

Our longitudinal data indicate that AMs are more likely to have better mental health than IMs, relapsers and adopters. This also applies perceived health and satisfaction with life, where AMs had higher odds for subjective good health and are more satisfied with their life compared to the other physical activity patterns.

The main strength of the present study is the ten-year follow-up from adolescence to young adulthood in a representative population-based sample. The study is also unique looking at ten-year physical activity patterns, the change of physical activity, assessing physical activity over several time points, and its impact on subsequent cardio-metabolic risk factors and mental health. However, we acknowledge there are some limitations. Physical activity is measured using validated questions, but relies on self-report, rather than objective measures. These physical activity questions have shown high reliability and acceptable validity [13,26], and dichotomisation as “active” and “inactive” provides good information on physical activity patterns. Another limitation could be that PA behavior might have changed very early in the ten-year period studied, obscuring the classification in some of the participants. Measuring physical activity four years after baseline, thus being able to see when the change of PA behaviour occurred both for adopters and relapsers (see Results section), revealed that nearly the same number of people changed their PA early and late in this ten-year period. We thus believe that this limitation is of minor importance.

Not having basal metabolic measurements in adolescence is a limitation because some of the participants could have CVD risk or mental distress at baseline, independent of their physical activity. Another limitation to our study may be the low participation rate at follow-up. HUNT Studies are based on repeated cross sectional studies, but as HUNT is a study of a total population longitudinal studies, as the follow-up from Young-HUNT1 to HUNT3, are possible. Although the participation rate in Young-HUNT 1 was high, the participation rate in HUNT 3 in the age group 20–29 years was low (31.5%). Many people in this age group had moved out of the county for further education or work and were not eligible for in vitiation to the HUNT 3 survey. Of the invited (5353 people), 42% of the women and 30% of the men participated. There were no significant differences between Young-HUNT1 participants who also participated at HUNT3 (follow-up) and those who did not regarding mean BMI, systolic and diastolic blood pressure, heart rate and physical activity. We therefore have strong reasons to believe that there are no major selection effects on physical activity or health behaviors between the two groups.

## Conclusion

Our study revealed a strong association between low physical activity and CVD risk and impaired mental health; those who maintained high physical activity from adolescence to young adulthood had a better CVD risk profile and better mental health status compared to people with physically inactive behaviour. Active maintainers had a significantly lower CVD risk, and less mental distress than those with other physical activity behaviours.

Adopting physical activity from adolescence to young adulthood may not necessarily confer a lower CVD risk, compared to inactive maintainers and relapsers, and may not be associated with better mental health outcomes. These data suggest that the multiple health benefits are attributable to behavioural maintenance of activity in adolescence, rather than any other pattern, such as behavioural adoption. These findings suggest that interventions on physical activity should start early in adolescence, and focus on maintaining physical activity to maximise health benefits a decade later. A key factor in public health strategies is that the promotion of physical activity needs to be kept a public health priority in the whole lifespan and with a special focus in the first 15 years of life.

## Competing interests

The authors declare that they have no competing interest.

## Authors' contributions

VR made a substantial contribution to the initial conception of the research reported in this paper, designing this study, analyzing, interpreting data and writing the manuscript. AB, KM and TLH made a substantial contribution to the conception of the research reported in this paper, reviewing drafts and interpreting data. The writing of the manuscript was led by VR, but all authors provided comments and contributed to the manuscript writing. All authors reviewed the manuscript critically and gave the final approval of the manuscript.

## Supplementary Material

Additional file 1 **Table S1.** Different physical activity patterns in relation to CVD risk in young adulthood (n=1869). Click here for file

Additional file 2 **Table S2.** Different physical activity patterns in relation to subsequent CVD risk (n=1072): comparing “adopters” to other Groups. Click here for file
