# Supplementary material for: Is physical activity maintenance from adolescence to young adulthood associated with reduced CVD risk factors, improved mental health and satisfaction with life: the HUNT Study, Norway
Source: Int J Behav Nutr Phys Act. 2012 Dec 14;9:144. doi: 10.1186/1479-5868-9-144 (PMC3541207; doi:10.1186/1479-5868-9-144)
Supplement: Additional file 1 — Table S1. Different physical activity patterns in relation to CVD risk in young adulthood (n=1869). [file 1479-5868-9-144-S1.pdf]

Additional file 1. Different physical activity patterns in relation to CVD risk in young adulthood (n=1869)

Variables	IMs against AMs*			IMs against AMs*			IM+relap+adopt against AMs#			IM+relap+adopt against AMs #		
	Unadjusted			Adjusted for age and gender			Unadjusted			Adjusted for age and gender		
	B	*P*	95% CI	B	*P*	95% CI	B	*P*	95% CI	B	*P*	95% CI
BMI	-.62	.035	-1.19, -0.5	-.44	.135	-1.02, 0.14	-.62	.004	-1.05, - 0.20	-.53	.015	-0.96, -0.10
Waist Circumference (cm)	-3.35	.000	-4.95, -1.75	-2.61	.001	-4.19, -1.03	-2.76	.000	-3.93, -1.59	-2.19	.000	-3.34, -1.04
Resting heart rate (HR)	-5.72	.000	-7.08, -4.35	-5.98	.000	-7.33, -4.63	-4.67	.000	-5.67, -3.66	-4.93	.000	-5.93, -3.93
Diastolic blood pressure (mmHg)	-1.47	.013	-2.62, -0.31	-1.00	.086	-2.15, 0.14	-1.30	.002	-2.13, -0.47	-.98	.019	-1.79, -0.16
Systolic blood pressure (mmHg)	-.68	.416	-2.32, 0.96	-.01	.994	-1.47, 1.46	-1.28	.035	-2.47, -0.09	-.50	.352	-1.54, 0.55
HDL-cholesterol (mmol/l)	.08	.000	0.04, 0.12	.06	.005	0.02, 0.09	.07	.000	0.04, 0.10	.05	.001	0.02, 0.08
Cholesterol (total) (mmol/l)	-.13	.031	-0.25, -0.01	-.10	.100	-0.22, 0.02	-.18	.000	-0.28, -0.09	-.18	.000	-0.27, -0.08
Glucose (mmol/l)	.04	.554	0.10, 0.19	.08	.308	-0.07, 0.23	.01	.853	-0.10, 0.13	.04	.533	-0.08, 0.15
Triglycerides	-.14	.035	-0.27, -0.01	-.10	.124	-0.23, 0.03	-.18	.000	-0.28, -0.08	-.15	.003	-0.25, -0.05

* Inactive maintainers (IMs) against active maintainers (AMs)

# inactive maintainers(IMs) + relapsers + adopters against active maintainers(AMs)

B = unstandardized regression coefficients
