# Supplementary material for: Is physical activity maintenance from adolescence to young adulthood associated with reduced CVD risk factors, improved mental health and satisfaction with life: the HUNT Study, Norway
Source: Int J Behav Nutr Phys Act. 2012 Dec 14;9:144. doi: 10.1186/1479-5868-9-144 (PMC3541207; doi:10.1186/1479-5868-9-144)
Supplement: Additional file 2 — Table S2. Different physical activity patterns in relation to subsequent CVD risk (n=1072): comparing “adopters” to other Groups. [file 1479-5868-9-144-S2.pdf]

Additional file 2. Different physical activity patterns in relation to subsequent CVD risk (n=1072): comparing "adopters" to other groups

Variables	Adopters against AMs* Unadjusted			Adopters against AMs* Adjusted for age and gender			Adopters against IMs + rel.# Unadjusted			Adopters against IMs + rel. # Adjusted for age and gender		
	B	*P*	95% CI	B	*P*	95% CI	B	P	95% CI	B	P	95% CI
BMI	-.70	.027	-1.33, -0.80	-.66	.036	-1.29, -0.04	-.11	.748	-0.78, 0.56	-.25	.465	-0.92, 0.42
Waist circumference (cm)	-1.90	.031	-3.62, -0.18	-1.87	.030	-3.56, -0.19	1.14	.222	-0.69, 2.98	.16	.861	-1.64, 1.97
Resting heart rate (HR)	-4.25	.000	-5.74, -2.76	-3.99	.000	-5.46, -2.51	.56	.482	-0.99, 2.10	1.12	.155	-0.42, 2.66
Diastolic blood pressure (mmHg)	-1.60	.011	-2.84, -0.37	-1.59	.011	-2.80, 0.37	-.40	.532	-1.65, 0.86	-.82	.196	-2.06, 0.42
Systolic blood pressure (mmHg)	-.73	.424	-2.53, 1.06	-1.27	.112	-2.83, 0.29	.73	.425	-1.07, 2.53	-.99	.225	-2.59, 0.61
HDL-cholesterol (mmol/l)	.03	.172	-0.01, 0.08	.04	.065	-0.00, 0.08	-.05	.036	-0.10, -0.00	-.00	.841	-0.05, 0.04
Cholesterol (total) (mmol/l)	-.21	.002	-0.35, -0.08	-.19	.004	-0.33, -0.06	-.04	.612	-0.19, 0.11	-.03	.690	-0.19, 0.12
Glucose (mmol/l)	.01	.929	-0.19, 0.21	.02	.857	-0.18, 0.22	-.00	.980	-0.17, 0.17	.06	.519	-0.23, 0.12
Triglycerides	-.12	.118	-0.27, 0.03	-.13	.073	-0.28, 0.01	.08	.297	-0.07, 0.24	.00	.938	-0.15, 0.16

Linear regression in separate models for each outcome

*Adopters against active maintainers (AMs)

Adopters against inactive maintainers (IMs) + relapsers

B = unstandardized regression coefficients
